# Endoscopic management of delayed post-papillectomy perforation

**DOI:** 10.1055/a-2695-4349

**Published:** 2025-09-24

**Authors:** Brian Lam, Sunil Gupta, Clarence Kerrison, Jun Young Kim, Hasib Ahmadzai, Nicholas Graeme Burgess, Michael J. Bourke

**Affiliations:** 1 8539 Department of Gastroenterology and Hepatology, Westmead Hospital, Sydney, Australia; 2 Westmead Clinical School, University of Sydney, Sydney, Australia

Ampullary adenomas are rare, with an annual incidence of 0.001%. En bloc endoscopic resection is recommended for lesions <30 mm without intraductal extension 1 .

A 78-year-old man underwent a papillectomy for a 25-mm ampullary adenoma. En bloc resection was achieved without evidence of deep mural injury. Clinical suspicion of a delayed perforation was raised due to persistent abdominal pain, fevers, and a rising C-reactive protein (359 mg/L) despite a normal white cell count (6.8 × 10 ^9^ /L) on day three post-resection. Despite a lack of oral contrast leak on computed tomography, the presence of gas locules and a small fluid collection around the duodenum ( Fig. 1 ) necessitated urgent endoscopic evaluation.

**Fig. 1:**
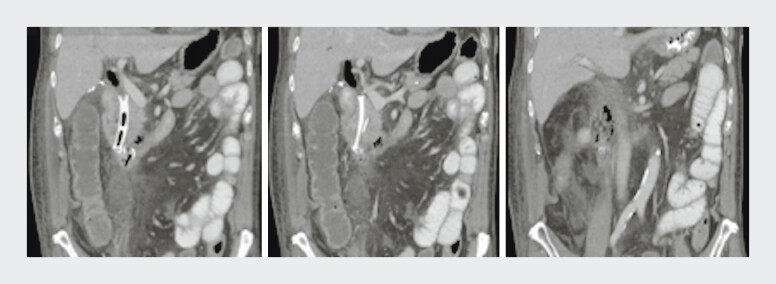
Computed tomography demonstrating small locules of retroperitoneal gas, fast stranding, and a small volume of extraluminal fluid.

Endoscopy identified a 10-mm perforation lateral to and distinct from the biliary orifice. Fluoroscopy confirmed contrast extravasation ( Fig. 2 ). The perforation was managed with three 16-mm through-the-scope (TTS) clips ( Video 1 ). One prong of each clip was placed into the distal bile duct and the other on the contralateral edge of the defect to maximize tissue capture and consequent closure strength. Complete luminal collapse via suctioning was achieved to facilitate tissue apposition before clip deployment. A fully covered self-expanding metal stent (10 mm × 6 cm) was deployed in the bile duct. Definitive closure was achieved.

**Fig. 2:**
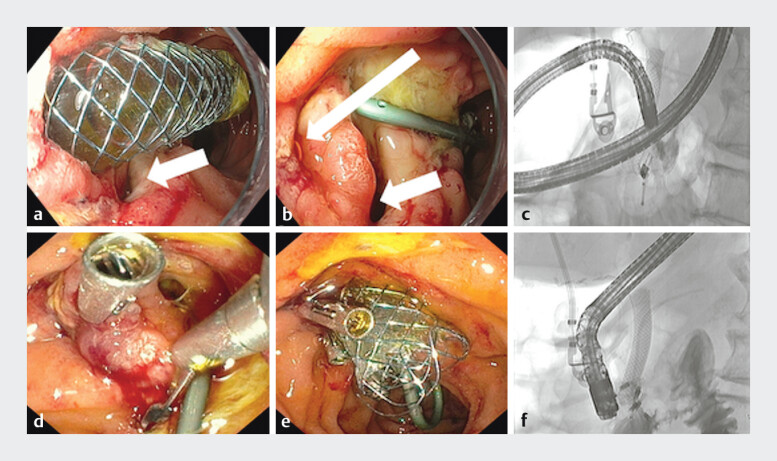
**a** Perforation noted as denoted by the arrow. **b** Perforation inspected carefully post-biliary stent removal with the biliary orifice denoted by the thin arrow, and the perforation by the thick arrow. **c** Perforation confirmed with fluoroscopy. **d** Clip closure of perforation with biliary cannulation prior to stent insertion. **e** Post-biliary stent insertion. **f** Fluoroscopic confirmation of complete closure.

Careful examination of the post-papillectomy perforation is demonstrated, followed by complete closure using through-the-scope clips and a biliary stent. Three-month follow-up confirms complete healing of the defect.Video 1

The patient remained nil by mouth for seven days before gradual diet upgrade prior to discharge. At the three-month follow-up, endoscopic reassessment confirmed complete mucosal healing.

This case highlights the importance of heightened vigilance after papillectomy for adverse events that may include perforation. Clinical features on serial exam were absent, but serial cross-sectional imaging showed progression in the radiological findings as well as corroborative blood results. The decision was made to endoscopically manage the perforation. To our knowledge this is the first case report of endoscopic management of a delayed post-papillectomy perforation.

Endoscopy_UCTN_Code_CPL_1AK_2AC

## Notes

Endoscopy E-Videos https://eref.thieme.de/e-videos
*E-Videos* is an open access online section of the journal *Endoscopy* , reporting on interesting cases and new techniques in gastroenterological endoscopy. All papers include a high-quality video and are published with a Creative Commons CC-BY license. Endoscopy E-Videos qualify for HINARI discounts and waivers and eligibility is automatically checked during the submission process. We grant 100% waivers to articles whose corresponding authors are based in Group A countries and 50% waivers to those who are based in Group B countries as classified by Research4Life (see: https://www.research4life.org/access/eligibility/ ). This section has its own submission website at https://mc.manuscriptcentral.com/e-videos .
